# Microfluidic Analyzer Enabling Quantitative Measurements of Specific Intracellular Proteins at the Single-Cell Level

**DOI:** 10.3390/mi9110588

**Published:** 2018-11-12

**Authors:** Lixing Liu, Beiyuan Fan, Diancan Wang, Xiufeng Li, Yeqing Song, Ting Zhang, Deyong Chen, Yixiang Wang, Junbo Wang, Jian Chen

**Affiliations:** 1 State Key Laboratory of Transducer Technology, Institute of Electronics, Chinese Academy of Sciences, Beijing 100190, China; liulixing16@mails.ucas.ac.cn (L.L.); fanbeiyuan@ucas.ac.cn (B.F.); lixiufeng13@mails.ucas.ac.cn (X.L.); 1141230132@ncepu.edu.cn (T.Z.); dychen@mail.ie.ac.cn (D.C.); 2 University of Chinese Academy of Sciences, Beijing 100190, China; 3 Peking University School of Stomatology, Beijing 10081, China; bjdxwdc@gmail.com (D.W.); duo.k.tong@gmail.com (Y.S.)

**Keywords:** instrumentation, microfluidic flow cytometry, intracellular proteins, absolute quantification

## Abstract

This paper presents a microfluidic instrument capable of quantifying single-cell specific intracellular proteins, which are composed of three functioning modules and two software platforms. Under the control of a LabVIEW platform, a pressure module flushed cells stained with fluorescent antibodies through a microfluidic module with fluorescent intensities quantified by a fluorescent module and translated into the numbers of specific intracellular proteins at the single-cell level using a MATLAB platform. Detection ranges and resolutions of the analyzer were characterized as 896.78–6.78 × 10^5^ and 334.60 nM for Alexa 488, 314.60–2.11 × 10^5^ and 153.98 nM for FITC, and 77.03–5.24 × 10^4^ and 37.17 nM for FITC-labelled anti-beta-actin antibodies. As a demonstration, the numbers of single-cell beta-actins of two paired oral tumor cell types and two oral patient samples were quantified as: 1.12 ± 0.77 × 10^6^/cell (salivary adenoid cystic carcinoma parental cell line (SACC-83), *n_cell_* = 13,689) vs. 0.90 ± 0.58 × 10^5^/cell (salivary adenoid cystic carcinoma lung metastasis cell line (SACC-LM), *n_cell_* = 15,341); 0.89 ± 0.69 × 10^6^/cell (oral carcinoma cell line (CAL 27), *n_cell_* = 7357) vs. 0.93 ± 0.69 × 10^6^/cell (oral carcinoma lymphatic metastasis cell line (CAL 27-LN2), *n_cell_* = 6276); and 0.86 ± 0.52 × 10^6^/cell (patient I) vs. 0.85 ± 0.58 × 10^6^/cell (patient II). These results (1) validated the developed analyzer with a throughput of 10 cells/s and a processing capability of ~10,000 cells for each cell type, and (2) revealed that as an internal control in cell analysis, the expressions of beta-actins remained stable in oral tumors with different malignant levels.

## 1. Introduction

Single-cell protein expressions provide key insights in studying cellular heterogeneities such as tumour heterogeneities and immune response variations [1,2,3]. Currently, flow cytometry is the golden instrument for single-cell protein analysis where cells stained with fluorescence-labelled antibodies rapidly travel through a capillary while the fluorescent intensities are measured [4,5]. Using calibration beads with the numbers of surface proteins under well controls, quantitative flow cytometry enables the absolute quantification of single-cell surface proteins while the copy numbers of intracellular proteins at the single-cell level remain elusive due to the lack of calibration approaches [6,7,8,9].

Microfluidics is an approach to processing fluids based on microfabricated channels (1–100 μm) [10,11], and due to their dimensional comparisons with biological cells, microfluidic instruments have been developed for single-cell protein analysis [12,13]. More specifically, barcoding microchips with a commercial brand of “Isoplexis” were developed where individual cells are confined within microchambers, and the absolute quantification of specific intracellular proteins is realized by cell lysis and the captures of target cellular proteins by preprinted antibodies on the bottom surfaces of the microchips [14,15,16,17]. In a second microfluidic approach, with a commercial brand of “single-cell westerns”, key steps of settling single cells into microwells, lysis in situ, gel electrophoresis, photoinitiated blotting to immobilize proteins, and antibody probing are included, enabling the quantitative analysis of cellular proteins [18,19,20]. However, in comparison to flow cytometry, these microfluidic instruments still suffer from limited throughputs since they cannot characterize single cells in a continuous fluid flow.

In order to address this issue, previously we developed a constriction channel-based microfluidic platform to quantify specific intracellular proteins of single cells in a high-throughput manner [21,22]. In this modified flow cytometry, cells stained with fluorescence-labelled antibodies are forced to deform through a constriction channel (a microfabricated channel with a cross-sectional area smaller than a cell), with raw fluorescent profiles collected. Meanwhile, solutions of fluorescent antibodies are flushed through this constriction channel to produce calibration curves, enabling the translation of raw fluorescent signals into copy numbers of specific intracellular proteins.

In this study, the instrumentation of the aforementioned approach was demonstrated, where the functionalities of individual modules were realized and the key parameters of the assembled instrument (e.g., detection resolutions, ranges, and throughputs) were characterized. In addition, the developed instrument was used to quantify numbers of single-cell beta-actins from two paired oral tumor cell types and two oral patient samples as a validation of the developed instrument. Furthermore, based on the results of this instrument, the expressions of beta-actins at the single-cell level remained stable within oral tumor cells, validating the use of beta-actins as internal controls in cell analysis. The structure of this paper is as follows: In Section 2, working mechanisms of the developed instrument are described. In Section 3, module functionalities, instrument operation, and data processing are described in detail. In Section 4, absolute quantification of beta-actins at the single-cell level are demonstrated. Conclusion and future work are included in Section 5.

## 2. Schematics and Working Mechanisms

**Figure 1:**
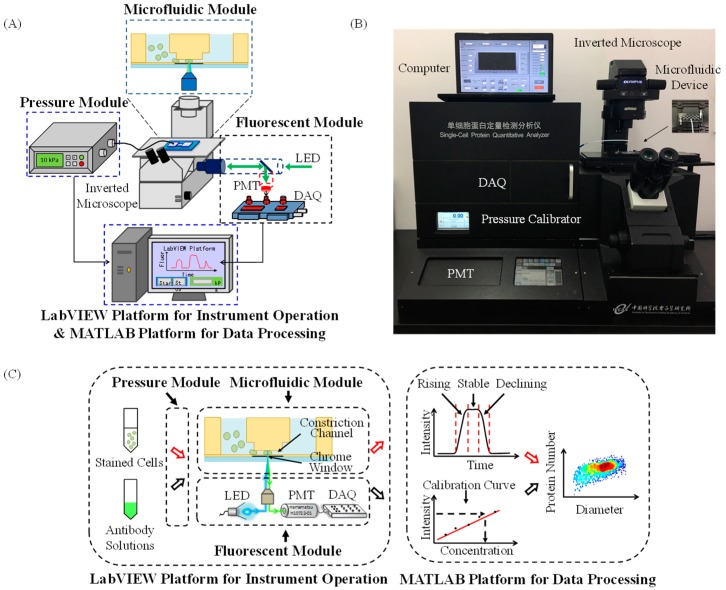
(**A**) Schematics and (**B**) a prototype of the fluorescent microfluidic flow cytometry enabling the measurement of copy numbers of specific intracellular proteins at the single-cell level. The developed instrument had five key components: A microfluidic module, a fluorescent module, a pressure module, a LabVIEW platform for instrument operation, and a MATLAB platform for data processing. (**C**) Working flow chart of the developed microfluidic instrument. Under the control of the LabVIEW platform for instrument operation, the pressure module flushed cells stained with fluorescence-labelled antibodies through the constriction channel of the microfluidic module while fluorescent intensities were quantified by the fluorescent module and then were further translated to the copy number of specific intracellular proteins at the single-cell level, leveraging the MATLAB platform for data processing.

Figure 1A,B shows schematics and a prototype of the microfluidic flow cytometry enabling the measurement of copy numbers of specific intracellular proteins at the single-cell level. The developed instrument had five key components, including a microfluidic module composed of a constriction channel with a microfabricated chrome window; a fluorescent module composed of an inverted microscope, a light source of a light-emitting diode (LED), a photomultiplier tube (PMT), and a data acquisition card (DAQ); a pressure module composed of a pressure controller; a LabVIEW platform (National Instruments, Austin, TX, USA) for instrument operation; and a MATLAB platform (The MathWorks, Inc., Natick, MA, USA) for data processing.

The flow chart of the developed flow cytometry is shown in Figure 1C. In operations, under the control of the LabVIEW platform for instrument operation, the pressure module flushed cells stained with fluorescence-labelled antibodies through the constriction channel of the microfluidic module. While the cell traveled through the fluorescent detection region defined by the constriction channel and the patterned chrome window, raw fluorescent signals were collected by the fluorescent module. Since the cross-sectional area of the constriction microchannel was smaller than cells, cells fully filled the constriction channel during their squeezing processes, and thus (1) cells traveled through the fluorescent detection region one by one, enabling single-cell protein quantification, and (2) solutions with fluorescence-labelled antibodies were flushed into the constriction channel directly, based on the pressure module, to form calibration curves.

Using the MATLAB platform for data processing, the fluorescent profile of a traveling cell through the fluorescent detection region of the microfluidic module could be divided into a rising domain, a stable domain, and a declining domain (see Figure 1C). More specifically, in the rising domain, there was a gradual increase in the fluorescent intensity, indicating that the deformed cell with fluorescence-labelled antibodies gradually filled the fluorescent detection region defined by the constriction channel and the patterned chrome window. As the deformed cell further moved in the constriction microchannel, there was a duration with the stable fluorescent intensity, which indicated the full occupation of the fluorescent detection region. As for the declining domain, it corresponded to the gradual decrease in fluorescent intensities in the leaving of the deformed cell. Based on this analysis, the diameters of cells and peaking values of fluorescent signals could be obtained through interpreting fluorescent profiles. Furthermore, leveraging the calibration curve, these raw parameters could be translated into the copy numbers of a targeted protein at the single-cell level. For detailed steps of pulse processing, please refer to previous publications [21,22].

## 3. Module Functionality, Operation, and Data Processing

### 3.1. Microfluidic Module

**Figure 2:**
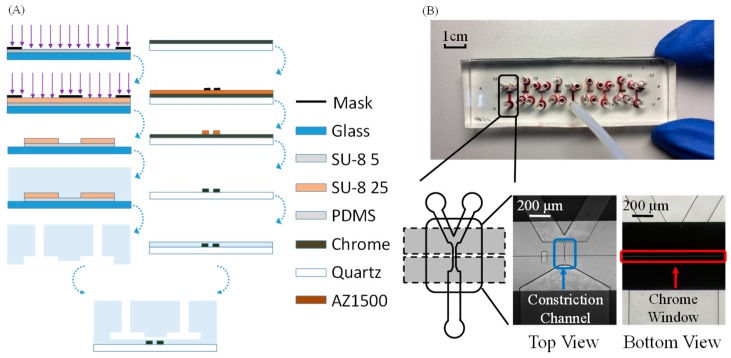
(**A**) Fabrication process and (**B**) a prototype device of the microfluidic module composed of a polydimethylsiloxane (PDMS)-based constriction channel with a patterned chrome window on quartz. The fabrication was based on conventional lithography including key steps of SU-8 exposure, PDMS molding, chrome patterning on quartz, and the bonding of the constriction channel layer and the chrome layer.

The microfluidic module consisted of a constriction channel with a cross-section area of 8 μm × 8 μm and a chrome window of 2.5 μm. The proposed device was fabricated based on conventional microfabrication techniques in which the patterned polydimethylsiloxane (PDMS, Dow Corning Corp., Midland, MI, USA) layer, including constriction channels, was replicated from a double-layer SU-8 (MicroChem Corp., Westborough, MA, USA) mould and bonded with a quartz slide that was first patterned with a chrome layer and then coated with a thin layer of unpatterned PDMS (see Figure 2A). For detailed information, please refer to the Supplementary Materials of this paper and previous publications [21,22]. The fabricated microfluidic devices are shown in Figure 2B, where multiple constriction channels could be fabricated in one single device.

### 3.2. Instrument Operation

**Figure 3:**
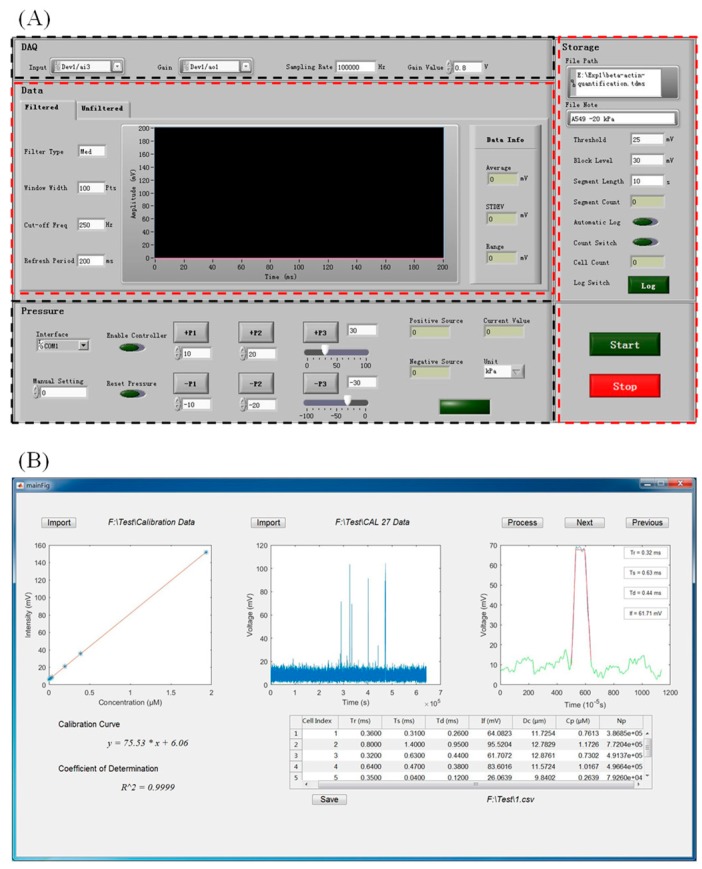
(**A**) The interface of the LabVIEW Platform for instrument operation mainly included the top-left and bottom-left areas for the regulations of the fluorescent and the pressure modules, respectively. The middle area displayed the sampled voltages collected by the data acquisition card (DAQ) in a real-time manner, and the right area displayed the parameters for the storage of the collected signals. (**B**) The interface of the MATLAB-based data processing mainly included the import of the calibration curve (left), the import of the raw voltage data indicating fluorescent intensities (middle), and the curve-fitting of individual fluorescent pulses (right). Based on these steps, the numbers of protein copies for individual cells were obtained and displayed at the bottom of this interface.

Figure 3A and Video I show the LabVIEW platform for instrument operation, which regulated the fluorescent and pressure modules. There were two key parameters in controlling the fluorescent module, the “gain voltage” of PMT (H10722-01, Hamamatsu, Japan) and the “sampling rate” of DAQ (PCI-6221, National Instruments, Austin, TX, USA) (see top-left of Figure 3A). An increase in the gain voltage of PMT could increase the amplification ratio of weak fluorescent signals, which at the same time increased background noises. After a balance between fluorescent amplifications and basal noise levels, in this study a gain voltage of 0.8 V was used, producing an amplification ratio of 3 × 10^5^ and a basal noise level of 5 mV. In addition, durations of travelling cells were several milliseconds, and thus a sampling rate of 100 kHz was used, enabling the collection of at least 100 points for individual fluorescent pulses. Note that the physical components of the fluorescent module also included the light source of LED (M470L3-C1, Thorlabs, Newton, NJ, USA) and an inverted microscope (IX 83, Olympus, Tokyo, Japan), which did not need controls from the LabVIEW-based operation platform.

In regulating the pressure module, the output pressure value generated by the pressure calibre (Druck PACE-5000, Burlington, VT, USA) was defined in the LabVIEW platform, with the real-time pressure values measured and displayed (see bottom-left of Figure 3A). In this instrument, high-value negative pressures were usually preferred for high-throughput analysis, and thus buttons for directly generating pressure values (e.g., −10 kPa, −20 kPa, and −30 kPa) were included in this interface. In addition, facing channel blockages, high-value positive pressures were also needed to blow away blocking particles, and thus buttons for directly generating pressure values (e.g., 10 kPa, 20 kPa, and 30 kPa) were also included in this interface.

Under the control of the LabVIEW platform for instrument operation, fluorescent and pressure modules were well coordinated to collect fluorescent signals of stained individual cells in a semiautomatic manner (see Video I Instrument Operation). In this video clip, each pulse represented a traveling cell, and the time durations for individual fluorescent pulses were between 1 and 10 ms. Thus, the throughput of this instrument was estimated at 10 cells/s when the time gaps among incoming cells were taken into consideration. Note that the throughput of this system was still two orders lower than the throughput of conventional flow cytometry since in this study, in order to improve signal-noise ratios of fluorescent intensities for travelling cells, a median filtration was conducted to process raw voltage signals collected by DAQ where fluorescent pulses with time durations lower than 0.5 ms were treated as noises and thus removed, limiting the further improvements in detection throughputs of this system.

### 3.3. Data Processing

Figure 3B and Video II show the MATLAB platform for semiautomatic data processing, which translated raw fluorescent signals into copy numbers of specific intracellular proteins. As shown in Video II Data Processing, in the first step, the calibration curve illustrating the relationship between concentrations of specific antibody and fluorescent intensities was loaded into the MATLAB platform. Then, raw fluorescent signals were loaded into the MATLAB platform and pulses were processed in a time sequence. Each pulse was first divided into a rising domain, a stable domain, and a declining domain, and then was processed to cell sizes and fluorescent intensities, which were further translated into copy numbers of specific intracellular proteins based on the calibration curve. Leveraging the MATLAB platform for data processing, it took ~10 min to process ~10,000 fluorescent pulses, realizing the quantitative measurements of specific intracellular proteins at the single-cell level. For detailed information on the processing of fluorescent pulses, please refer to previous publications [21,22].

## 4. Demonstration

Based on the calibration curves of three types of fluorescence-labelled macromolecules, the detection ranges and resolutions of the proposed instrument were first characterized. More specifically, the detection ranges and resolutions of Alexa 488, FITC, and FITC-labelled anti-beta-actin antibodies were quantified as 896.78–6.78 × 10^5^ and 334.60 nM, 314.60–2.11 × 10^5^ and 153.98 nM, and 77.03–5.24 × 10^4^ and 37.17 nM, respectively. These results indicated that in comparison to Alexa 488, the same numbers of FITC molecules could produce higher fluorescent intensities. As to the comparison of FITC and FITC-labelled anti-beta-actin antibodies, since there were multiple FITC molecules for each antibody, the detection resolution for FITC-labelled anti-beta-actin antibodies was much lower than for FITC.

As a functional demonstration, the developed microfluidic instrument was used to characterize the numbers of beta-actins at the single-cell level. Note that as an internal control in cell analysis, the expressions of beta-actins were assumed to be stable within individual cells, which was questionable due to recently reported quantitative results at the single-cell level [23].

**Figure 4:**
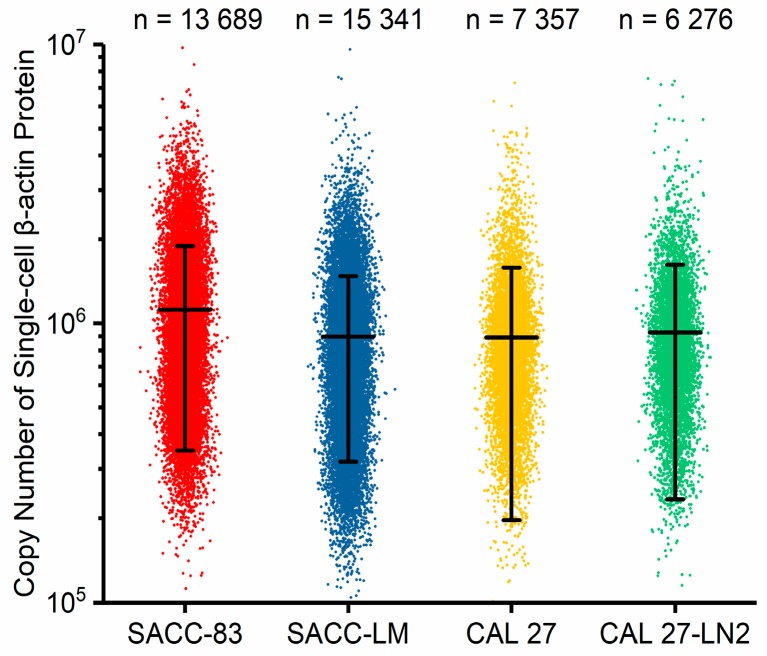
Scatter plots of the copy numbers of single-cell β-actin proteins for salivary adenoid cystic carcinoma parental cell line (SACC-83) (*n_cell_* = 13,689), salivary adenoid cystic carcinoma lung metastasis cell line (SACC-LM) (*n_cell_* = 15,341), oral carcinoma cell line (CAL 27) (*n_cell_* = 7357), and oral carcinoma lymphatic metastasis cell line (CAL 27-LN2) (*n_cell_* = 6276). These results indicated that the developed instrument was capable of collecting beta-actins from ~10,000 single cells.

In order to address this issue, in this study two paired oral tumor cell types were characterized, where single-cell beta-actin numbers were obtained as 1.12 ± 0.77 × 10^6^/cell (salivary adenoid cystic carcinoma parental cell line (SACC-83), *n_cell_* = 13,689) versus 0.90 ± 0.58 × 10^6^/cell (salivary adenoid cystic carcinoma lung metastasis cell line (SACC-LM), *n_cell_* = 15,341), and 0.89 ± 0.69 × 10^6^/cell (oral carcinoma cell line (CAL 27), *n_cell_* = 7357) versus 0.93 ± 0.69 × 10^6^/cell (oral carcinoma lymphatic metastasis cell line (CAL 27-LN2), *n_cell_* = 6276) (see Figure 4 and Supplementary Materials for cell culture and preparation).

These experimental results validated the developed microfluidic platform, which could collect the numbers of beta-actins from ~10,000 cells in total for each cell type. Meanwhile, the values of quantified single-cell beta-actins were around 1 × 10^6^/cell for these four cell types, whereas no significant differences were located for oral tumor cells with different malignant levels. Thus, these results indicated that the expression levels of beta-actins at the single-cell level remained stable for oral tumor cell types.

**Figure 5:**
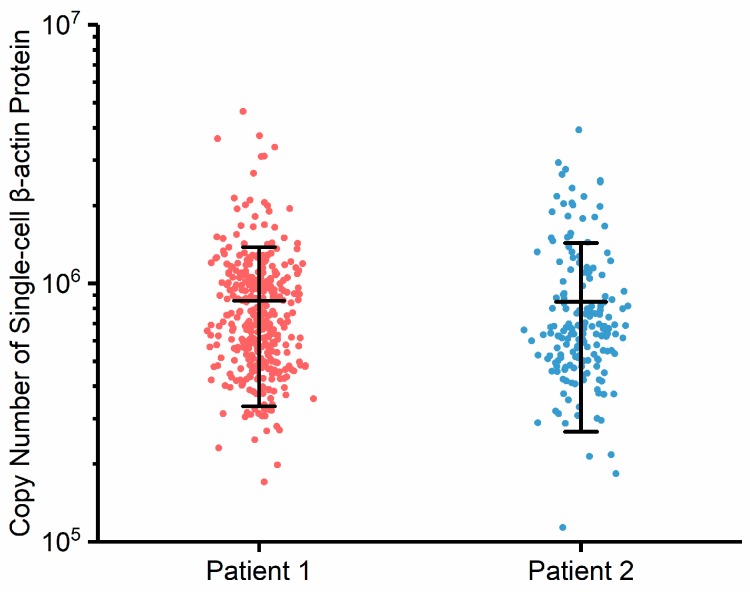
Scatter plots of the copy numbers of single-cell β-actin proteins from two oral tumor patients. These results indicated that the developed instrument could be used to absolutely quantify specific intracellular proteins of patient samples at the single-cell level.

Furthermore, the developed microfluidic instrument was used to characterize two patient samples of oral tumors, where single-cell beta-actin copy numbers were quantified as 0.86 ± 0.52 × 10^6^/cell (*n_cell_* = 365) and 0.85 ± 0.58 × 10^6^/cell (*n_cell_* = 177) (see Figure 5 and Supplementary Materials for cell preparation and data processing). These results further validated the functionality of the developed instrument, which was capable of processing real clinical samples. Furthermore, the quantified numbers of beta-actins of these two oral tumor samples were comparable with the results of oral tumor cell lines of CAL 27, confirming again that the expression levels of beta-actins at the single-cell level remained stable within oral tumor cells. Note that in processing clinical samples, the total numbers of characterized cells were two orders lower than the counterparts of cultured cell lines. In comparison to experiments with cell lines, clinical samples were noticed to block the constriction channels more often, limiting the processing throughput. Since this blockage may have resulted from cell clusters in clinical samples, in future studies size-based filtrations are recommended to remove cell aggregations before the clinical samples are flushed into this microfluidic platform.

In comparison to previous papers [21,22], where the pressure sources and fluorescent detections were controlled manually, the LabVIEW-based platform developed in this study enabled the system to function in a continuous manner and collect data from ~10,000 cells for each cell type with much lower experimental durations. In addition, the semiautomatic platform functioned in a robust manner and processed the real clinical samples, which was not reported in previous publications. Furthermore, in comparison to previous papers [21,22] where the fluorescent pulses were processed manually, the MATLAB platform for data processing developed in this study enabled the semiautomatic processing of fluorescent pulses, which significantly decreased the time durations of data processing.

## 5. Conclusions and Future Work

In this study, the instrumentation of a microfluidic analyzer was demonstrated, which enabled the measurement of single-cell numbers of specific intracellular antibodies at a throughput of roughly 10 cells/s. The functionalities of individual modules were validated and integrated to form the prototype instrument, where the numbers of beta-actins of two paired oral tumor cell types and two samples of oral tumor patients were collected and compared. Future developments of the instrument are aimed at the absolute quantification of multiple types of intracellular proteins simultaneously (e.g., beta-actins, alpha-tubulins, and beta-tubulins) at a higher throughput (i.e., 1000 cells/s).

## Supplementary Materials

The following are available online at http://www.mdpi.com/2072-666X/9/11/588/s1.
