# Supplementary material for: Microfluidic Analyzer Enabling Quantitative Measurements of Specific Intracellular Proteins at the Single-Cell Level
Source: Micromachines (Basel). 2018 Nov 12;9(11):588. doi: 10.3390/mi9110588 (PMC6265747; doi:10.3390/mi9110588)
Supplement: Supplementary file 1 [file micromachines-09-00588-s001.zip › micromachines-379120-SI/micromachines-379120-Supplementary Materials.doc]

### Supplementary Materials

**Title**

Device Fabrication and Cell Preparation

**Authors**

Lixing Liu^1,2^*, Beiyuan Fan^1,2^*, Diancan Wang^3^*, Xiufeng Li^1,2^, Yeqing Song^4^, Ting Zhang^1,2^, Deyong Chen^1,2^, Yixiang Wang^4^**, Junbo Wang^1,2^** and Jian Chen^1,2^**

**Affiliations**

^1^State Key Laboratory of Transducer Technology, Institute of Electronics, Chinese Academy of Sciences, Beijing 100190, China;

^2^University of Chinese Academy of Sciences, Beijing 100190, China;

^3^Department of Oral and Maxillofacial Surgery, Peking University School and Hospital of Stomatology, Beijing 10081, China;

^4^Yixiang Wang, Central laboratory, Peking University School and Hospital of Stomatology, Beijing 10081, China

*Co-First Authors

**Co-Corresponding Authors:

Yixiang Wang, Central laboratory, Peking University School and Hospital of Stomatology, E-mail: [kqwangyx@bjmu.edu.cn,](mailto:kqwangyx@bjmu.edu.cn,) Tel:+86-10-82195537

Junbo Wang, Institute of Electronics, Chinese Academy of Sciences, E-mail: [jbwang@mail.ie.ac.cn](mailto:jbwang@mail.ie.ac.cn), Tel/Fax: +86-10-58887191

Jian Chen, Institute of Electronics, Chinese Academy of Sciences, E-mail: [chenjian@mail.ie.ac.cn](mailto:chenjian@mail.ie.ac.cn), Tel/Fax: +86-10-58887256

**Materials**

All cell-culture reagents were purchased from Life Technologies Corporation (USA). Materials required for device fabrications included SU-8 photoresist (MicroChem Corp., USA) and 184 silicone elastomer (Dow Corning Corporation, USA).

More specifically, materials in cell isolation, culture and staining include collagenase/hyaluronidase/dispase solution (GIBICO, Life Technologies Corporation, USA) for tumor cell isolation from patient samples suffering from oral squamous cell carcinoma, DMEM medium (GIBICO, Life Technologies Corporation, USA), fetal bovine serum (GIBICO, Life Technologies Corporation, USA), penicillin and streptomycin (GIBICO, Life Technologies Corporation, USA), trypsin (GIBICO, Life Technologies Corporation, USA), phosphate buffer saline (GIBICO, Life Technologies Corporation, USA), FITC labelled anti-β-actin antibody (ABCAM, ABCAM Corporation, USA), paraformaldehyde (Sigma, Sigma-Aldrich Corporation, USA), triton x-100 (Sigma, Sigma-Aldrich Corporation, USA), and bovine serum albumin (Sigma, Sigma-Aldrich Corporation, USA).

**Device Fabrication**

The proposed device was fabricated based on conventional microfabrication including SU-8 mould fabrication, PDMS replication, chrome layer patterning and bonding (see Figure 2 of the manuscript). Briefly, the first layer of SU-8 forming the constriction channel (8 μm, SU-8 5) was spin coated on a glass substrate, followed by soft bake, exposure and post exposure bake without development. The second layer of SU-8 forming the cell loading channels (25 μm, SU-8 25) was spin coated on the first layer of SU-8, exposed with alignment and developed. PDMS prepolymer and curing agents (10:1 by weight) were mixed, degassed, poured on channel masters and cross-linked (overnight at 80 ºC in an oven). After full crosslinking, the PDMS replicas were then peeled from the SU-8 masters with inlets and outlets punched through.

The chrome window was fabricated by hard lithography, including steps of chrome deposition, photoresist patterning, chrome etching and coating of an ultra-thin PDMS layer (~1 μm) without patterning where PDMS prepolymer and curing agents (10:1 by weight) were dissolved in hexane (1:16), thoroughly mixed, degassed, and spin coated and cross-linked. In the end, after plasma treatment, the patterned PDMS layer and the quartz slide with the patterned chrome layer were bonded together.

**Human Specimen Isolation and Cell Culture**

Oral squamous cell carcinoma samples were obtained from patients undergoing surgical removal of tumor tissues, which were obtained with the agreement of the patients (please refer to the supplementary material of “Informed Consent Forms”) and approved by the Peking University School of Stomatology Medical Ethics Committee (#PKUSSIRB-201418116a). The methods involved with human specimen isolation and cell culture were carried out in accordance with the approved guidelines.

More specifically, isolated human samples were firstly thoroughly washed by PBS to remove blood residues. The samples were then cut into ~1 mm^3^ pieces and soaked within 1 mL of an enzyme solution (collagenase/hyaluronidase/dispase mixture) for about 4-6 hours at 37 °C with low speed agitations to facilitate enzymatic digestions. Following the digestion step, 5 mL DMEM was added to terminate the digestion process. Then the samples were centrifuged at 1000 rpm for 10 minutes and the cell pellets were resuspended and passed through a 40 μm cell strainer to obtain single-cell suspensions for intracellular staining.

All cell lines were purchased from China Infrastructure of Cell Line Resources and cultured in a cell incubator (3111, Thermo Scientific, USA) at 37°C in 5% CO_2_. More specifically, paired salivary adenoid cystic carcinoma cell lines of SACC-83 (parental cells) and SACC-LM (lung metastasis cells) and paired oral carcinoma cell lines of CAL 27 (parental cells) and CAL 27-LN2 (lymphatic metastasis cells) were cultured with DMEM media supplemented with 10% FBS and 1% penicillin and streptomycin, respectively. Prior to experiments, cells in logarithmic phases were trypsinized, centrifuged and resuspended in phosphate buffer saline with 0.5% bovine serum albumin at a concentration of ~10 million cells per mL.

**Intracellular Staining**

Intracellular staining of β-actin proteins was conducted, following well-established protocols used in conventional flow cytometry, which included key steps of fixation, membrane permeabilization, blocking and antibody staining. More specifically, firstly, the cell suspension was mixed with a 2% formaldehyde solution and incubated for 15 min at 4 ºC for fixation. Then, triton x-100 (e.g., 0.07% for SACC-83, 0.02% for SACC-LM, 0.05% for CAL 27, 0.05% for CAL 27-LN2, 0.05% for oral tumor cells isolated from tumor samples) was added for an incubation of 15 min at 4 ºC in order to penetrate cellular membranes. Then blocking was conducted based on 5% bovine serum albumin for 30 min at room temperature. FITC labelled anti-β-actin antibodies (1:100) were used to stain cells in suspension for 4 hours at 37 ºC for comparison.
